# The anti-cancerous activity of recombinant trichosanthin on prostate cancer cell PC3

**DOI:** 10.1186/s40659-016-0081-8

**Published:** 2016-03-25

**Authors:** JinLong Li, Hui Li, ZhaoLi Zhang, NianYue Wang, YongChen Zhang

**Affiliations:** Department of Laboratory Medicine, The Second Affiliated Hospital of Southeast University, Zhongfu Road 1-1, Nanjing, 210003 China; Department of Biochemistry and State Key Laboratory of Pharmaceutical Biotechnology, Nanjing University, Nanjing, 210093 China; Department of Neonatology, The Taizhou People’s Hospital, Taizhou, 225300 China; Department of Pharmacy, The Second Affiliated Hospital of Southeast University, Nanjing, 210003 China

**Keywords:** Trichosanthin, Interleukin IL-2, Prostate cancer cells PC3, Tumor

## Abstract

**Context:**

Trichosanthin produced in the root tube of *Trichosanthes kirilowii* shows anti-tumor activity on a series of cancer cells including Hela, MCF-7, HL-60. But there is little information about its effect on the carcinogenesis of prostate cancer.

**Objective:**

This work was designed to study the role of trichosanthin on prostate cancer cells PC3.

**Materials and methods:**

Trichosanthin was expressed in BL21 strain and purified by affinity chromatography. MTT assay was designed to determine the effect of trichosanthin on growth of PC3 cells at doses of 10, 20, 40, 60, 80, and 120 μg/ml. Then the effect of 50 μg/ml rTCS alone or combined with 2 μM IL-2 on PC3 cell proliferation was analyzed. And the mechanism of rTCS was studied by western blot. After that the in vivo effect of rTCS combined with IL-2 was explored in mice bearing PC3 xenograft tumor.

**Results:**

Trichosanthin was successfully expressed in BL21 and purified by 100 mM imidazole. It was shown to inhibit proliferation of PC3 cells in a dose-dependent manner with IC50 50.6 μg/ml. When combined with cytokine IL-2, a significant synergic effect was obtained. The inhibition rate on PC3 was around 50 % in combination group while only 35.5 % in single rTCS group at 50 μg/ml. Further, the expression of full length caspase-8 and Bcl-2 decreased significantly while cleaved caspase-8 and Bax were up-regulated, which suggest that caspase-8-mediated apoptosis pathway may be activated by rTCS in PC3 cells. Moreover, our data demonstrated that tumor volume and tumor weight were significantly reduced in rTCS-treated or rTCS/IL-2-treated nude mice bearing PC3 xenograft tumor compared with control. And significant difference was also found between rTCS and rTCS/IL-2 group.

**Conclusions:**

This study demonstrates that rTCS is a potential agent with high in vitro and in vivo anti-tumor activity on PC3 cells. And rTCS combined with IL-2 is a promising strategy in treating patients with prostate cancer in future.

## Background

Trichosanthin (TCS), extracted from the root tube of *Trichosanthes kirilowii,* is a single-chain protein which could inhibit protein synthesis by hydrolyzing N–C glycosidic bond of eukaryotic ribosome 28S rRNA [1]. TCS was used as an abortifacient drug to treat hydatidiform moles, invasive moles, and ectopic pregnancy [2, 3]. Empirical studies have attributed these effects to trichosanthin (hereafter referred to as RTCS), a 27-kDa protein purified from *T. kirilowii* tubers. TCS was reported to have immune-regulatory activity. It could ablate the replication of human immunodeficiency virus (HIV) and herpes simplex virus type 1 (HSV-1) [4, 5]. TCS is a potential antidote against some tumors as it could suppress the tumor cell growth in different ways. First, it can produce reactive oxygen species (ROS) and initiate apoptosis in human chorio-carcinoma (Jar) cells [6, 7]. Second, TCS can induce the death of human cervical carcinoma (Hela) cells by increasing cytosolic calcium accompanied with the suppression of cAMP/protein kinase C level [8, 9]. Third, the apoptosis-inducing activity of TCS applies to mouse NIH 3T3 embryonic fibroblasts by the activation of caspase-8 and caspase-3 pathways [10]. Fourth, TCS can induce apoptosis of human lung cancer cells by G1 phase arrest, anti-telomerase effects, and inhibition of cell migration and metastasis [11, 12], Furthermore, TCS was shown to elicit apoptosis of human HL-60 cells through caspase-3-mediated pathway [13, 14].

Prostate cancer is one of the most common diseases for men and remains a leading cause of death in most developed countries, especially for old men [15, 16]. Among all men diagnosed with cancer with age of over 70 years, about 50 % were prostate cancer. Although localized prostate cancer could be removed by surgery and radiation therapy, about 30–50 % patients would have a local or distant recurrence. So it is urgent to develop more potent drug or effective strategy to treat prostate cancer. IL-2 is a well-known molecule with activity of suppressing tumor cell growth [17, 18]. And IL-2 has been successfully applied in treating a series of tumor cells in combination with other agent [19, 20].

In this study, we found that recombinant trichosanthin (rTCS) could significantly inhibit proliferation of prostate cancer cells PC3 both in vitro and in vivo. And IL-2 could obviously enhance the inhibitory effect of rTCS on PC3 cell. Additionally, the caspase8-mediated apoptosis pathway was shown activated in PC3 cell. Our work broadened the medicinal applications of rTCS and the combination of rTCS with IL-2 serves as a novel strategy against human prostate cancer.

## Methods

### Cell lines and reagents

Cell line PC3 was a gift from Prof. Hedy and cultured in RPMI-1640 medium with 10 % fetal bovine serum (Invitrogen Co., USA) containing 100 μg/mL penicillin and 100 U/mL streptomycin (Biotech Biology Company). IL-2 was purchased from Bioscience Company. Ni Sepharose 6 Fast Flow was from GE healthcare (USA). Restriction enzymes (NdeI and NheI), Pfu DNA polymerase and T4 DNA ligase were purchased from Takara Biotech Co., Ltd. (Dalian, China).

### Construction of plasmid pET22b-rTCS

The primers PF and PR for rTCS was designed and added with NdeI and NheI restriction enzyme sites (Table 1). rTCS gene was amplified from plasmids pbs-rTCS preserved in our laboratory. The DNA fragment was digested with restriction enzymes, and cloned between NdeI and NheI sites of pMD18T-rTCS. The rTCS gene was confirmed by DNA sequencing. The PET22b vector and the rTCS gene were ligated by T4 ligase. The resulted plasmid, named PET22b-rTCS, was transformed into *E. coli* BL21(DE3) (TIANGEN Biotech Co., Ltd, China) following the standard procedure.

**Table 1:** Primers used for amplification of TCS

Primer	Sequence 5-3	Enzyme site added
Forward	cCATATGatgatcagattcttagtcctc	NdeI
Reverse	gGCTAGCctaaatagcataacttcca	NheI

### Expression and purification of recombinant rTCS

Overnight culture of *E. coli* BL21 (DE3) cells from a single colony were inoculated at ratio of 1 % (volume) into Luria–Bertani medium, and then grown at 37 °C for 3 h. IPTG was added to a final concentration of 0.4 mM at 25 °C for 8 h of induction until the medium was 0.6–0.8 of OD600. Cells were collected by centrifugation at 3500 rpm for 10 min, and then suspended in 50 ml cold lysis buffer and disrupted by sonication. The cell lysates were then centrifuged at 13,000 rpm for 10 min at 4 °C. The supernatant and pellet were subjected to a 12 % sodium dodecyl sulfate–polyacrylamide gel electrophoresis (SDS-PAGE). In brief, they were mixed with equal volume of 2 × SDS sample loading buffer and water respectively. After boiled for 5 min each sample was separated by 12 % separating gel. The gel was run at constant voltage of 120 V. After electrophoresis, SDS-PAGE gels were stained with Coomssie Brilliant Blue G-250.

Lysates were clarified by centrifugation and allowed to bind to nickel affinity resin for 1 h at 4 °Cwith gentle mixing. After being washed with lysis buffer, the rTCS protein was eluted with elution buffer solution (about 100 mM imidazole). The elution fractions were purified by nickel column with automated protein separation chromatography system.

### Western blot analysis of recombinant rTCS

Five microgram per millilitre protein was separated on 10 % SDS–polyacrylamide gel electrophoresis, and transferred to polyvinylidene fluoride (PVDF) membranes. Then the transferred PVDF was blocked at room temperature for 2 h in 5 % (w/v) nonfat milk in TBST (20 mM Tris–HCl, pH 7.6, 500 mM NaCl, 0.1 % Tween 20), and incubated overnight at 4 °C with the primary antibody in TBST buffer with 1 % (w/v) bovine serum albumin. After that PVDF was incubated with anti-trichosanthin antibody (Santa Cruz, USA). After washing with TBST, the membranes were incubated with goat-anti-mouse secondary antibodies (1:200) and determined with the ECL kit (Pierce Biotechnology, Rockford, IL, USA) according to the manufacturer’s instructions.

### Cell proliferation assay

The PC3 cells were seeded into 96-well microtiter plates at 1 × 10^4^ cells/well and incubated for 24 h. After treatment with various concentrations of recombinant rTCS (10, 20, 40, 60, 80, 120 μg/ml) and rTCS (50 μg/ml) +IL-2 (2 µM) for 48 h, cell viability assays were performed using the MTT method. MTT dissolved in PBS was added to the cultures at a final concentration of 0.5 µg/ml. After further incubation at 37 °C for 4 h, the medium was carefully removed and formazan crystals were dissolved in 150 μl DMSO per well, and the absorbance at 490 nm was measured on a pla

te reader with DMSO as a blank control. The cell inhibition rate was calculated as follow:

$${\rm Inhibition}\,\,{\rm rate} \,({\rm IR}) =({\rm A}490 \,\,{\rm of}\,\, {\rm untreated} \,\,{\rm samples}-{\rm A}490\,\,{\rm of}\,\,{\rm treated}\,\,{\rm samples})/{\rm A}490\,\,{\rm of}\,\,{\rm untreated}\,\,{\rm samples}\times100\,\%.$$

Data was analyzed from three independent experiments.

### In vivo xenograft studies

Eighteen female BALB/c nude mice (7 weeks old, 25 g) were obtained from the Model Animal Research Center of Nanjing University and procedures of all animal experiments were approved by the Nanjing University Animal Research Ethics Committee. The xenograft model was constructed as follows: 1 × 10^7^ PC3 cells in 200 μL RPMI 1640 medium were injected subcutaneously into the right flanks of mice. Once the tumors were about 100 mm^3^, mice were randomly divided into three groups (six mice per group). One group was treated with an intraperitoneal injection of rTCS at a dose of 5.0 mg/kg/day for 10 days. The other group was treated with rTCS (5.0 mg/kg/day) + IL-2 (1 mg/kg/day). In the control group, the same volume of PBS was administered. Tumor diameters were serially recorded with an electronic caliper every 2 days, and tumor volume was calculated with the formula:

$${\rm tumor}\,\,{\rm volume}\, ({\rm mm}^3)=0.5\times {\rm length}\times {\rm width}^2$$

Tumor body weight was weighed after mice were sacrificed on the 15th day.

### Statistical analysis

The IC50 value was calculated by SPSS11.0 software. All the data are shown in mean standard deviation (S.D.) from three independent experiments. Student t test was applied for the comparison between two groups. P value less than 0.05 was considered statistically significant.

## Results

### The expression and purification of rTCS protein

**Fig. 1:**
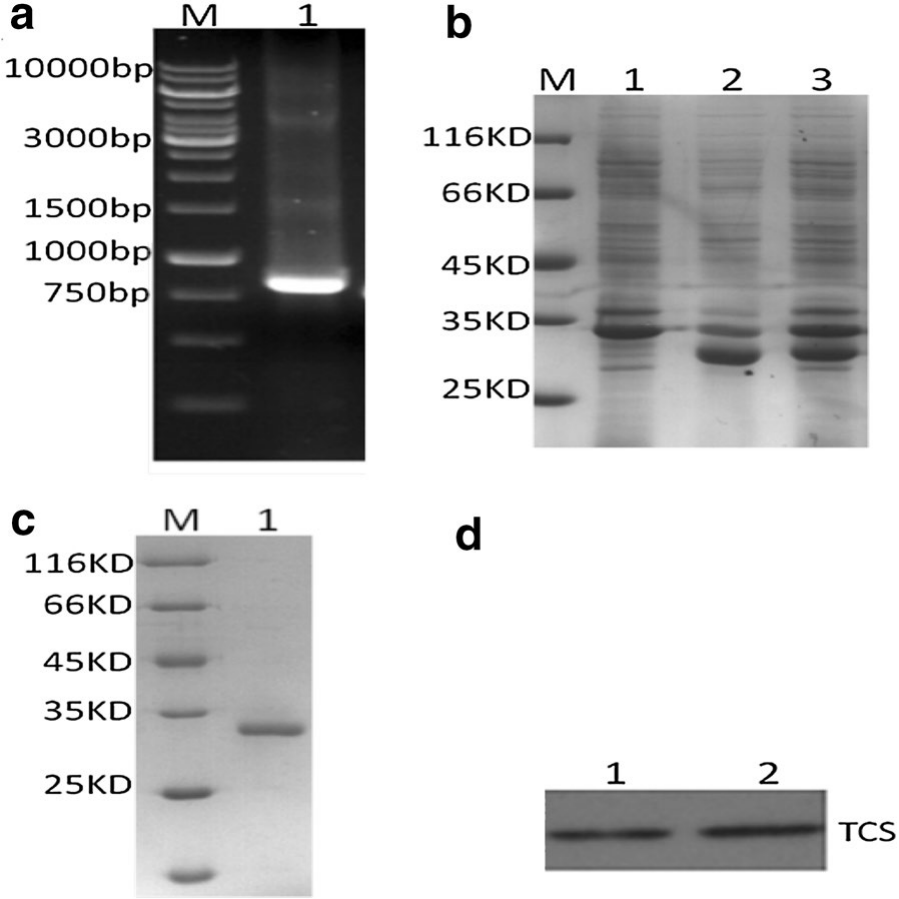
The expression and purification of rTCS. **a** PCR amplification of CDS for rTCS. *Lane 1* stands for the CDS coding rTCS. **b** Expression of rTCS in BL21 *E. coli.* *Lane 1* to *lane 3* stands for the control, clone 1, clone 2 respectively. **c** Purified rTCS by affinity adsorption. *Lane 1* stands for purified rTCS. D. Western blot analysis of rTCS with anti-TCS antibody. *Lane 1* and *lane 2* stand for the same sample

As shown in Fig. 1a, the coding sequence for rTCS was amplified and correctly cloned into expression vector pET22b. Then rTCS protein was expressed and purified as stated above. The optimized condition for rTCS expression was 4 h after induction with 0.4 mM IPTG (Fig. 1b). And 100 mM imidazole was used to purify rTCS (Fig. 1c). Western blot analysis with anti-rTCS monoclonal antibody further proved the band was rTCS (Fig. 1d). And as reported in early research, rTCS here was about 27.5 KDa.

### rTCS inhibited proliferation of PC3 cells

**Fig. 2:**
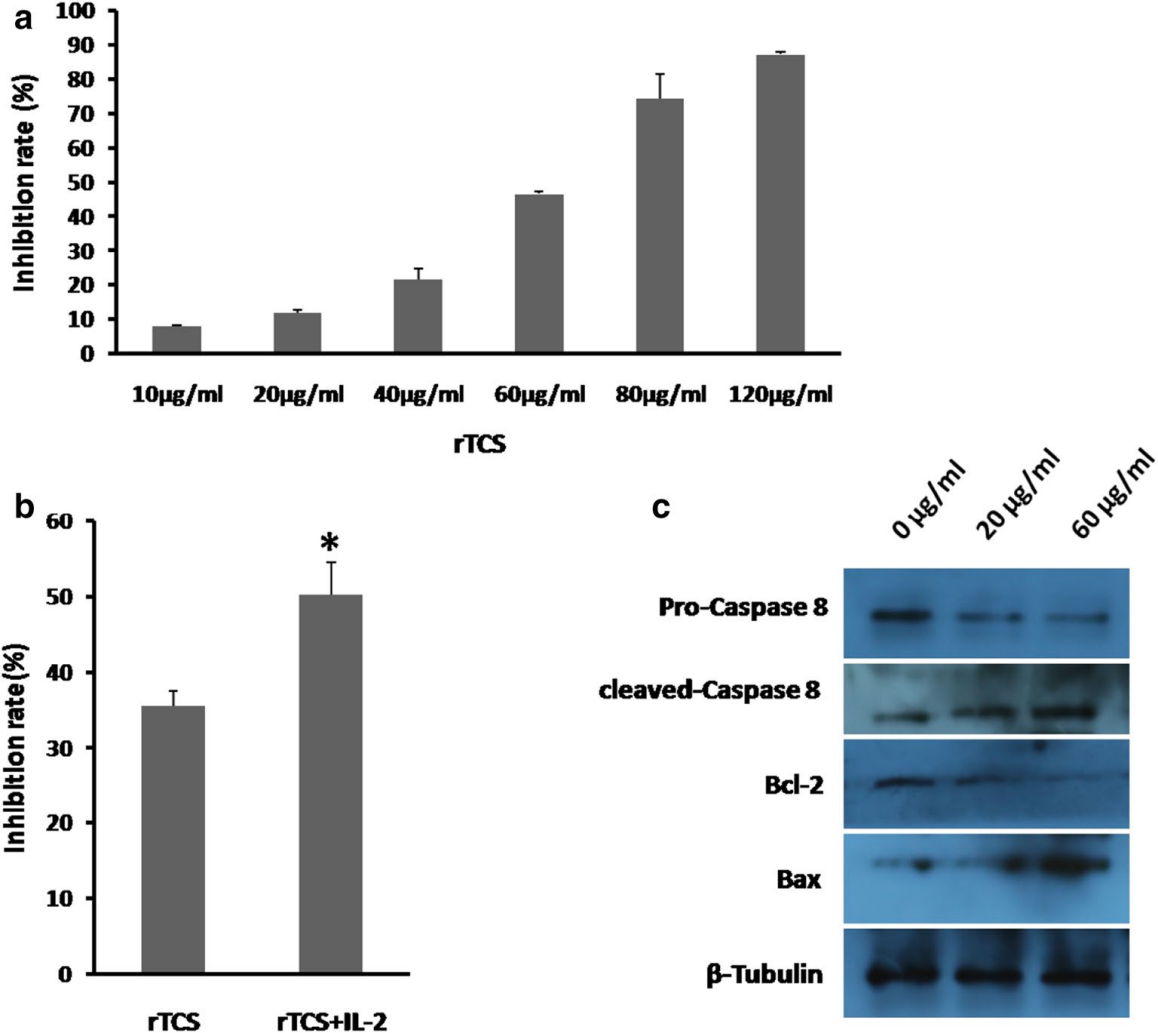
rTCS inhibits proliferation and induces apoptosis of PC3 cells. **a** A series of concentration of rTCS including 10, 20, 40, 60, 80, 120 ug/ml was used to treat PC3 cells. And rTCS was shown to inhibit proliferation of PC3 cells in a dose-dependent manner. **b** 50 µg/ml rTCS was combined with 2 μM IL-2 to treat PC3 cells. The inhibitory rate in combinatory group was about 50 % while it was only 35.5 % in 50 µg/ml rTCS single group. **c** rTCS activates caspase-8-mediated apoptosis. The level of full length caspase-8 and Bcl-2 decreased significantly in 20 or 60 µg/ml rTCS-treated group after 24 h. But the level of cleaved caspase-8 as well as Bax dramatically increased. Statistical analysis of data was performed with t-test, and *P<0.05 indicated a statistically significant difference

rTCS was reported to inhibit growth of several kinds of tumor cells including Hela, HL-60, lung cancer cells in early research. We demonstrated that rTCS could also suppress the growth of prostate cancer cells PC3. As shown in Fig. 2a, the growth of PC3 cells was significantly inhibited in rTCS-treated group compared to control. And this effect was dose dependent which was in consistent with the results on breast cancer cells MCF-7. The IC_50_ was about 50.6 µg/ml. IL-2 was a well-known cytokine with activity of suppressing tumor cell growth. And IL-2 has been successfully applied in clinic to treat a series of tumors. In this study, we proved that IL-2 could obviously enhance the inhibitory effect of rTCS on PC3 cell. As in Fig. 2b, the inhibition rate of PC3 cell in rTCS/IL-2-treated group was about 50 % while it was only 35.5 % in rTCS-treated group alone, which suggest that there is an obvious synergic effect between rTCS and IL-2.

### rTCS activated caspase-8-mediated apoptosis in PC3 cells

Evading apoptosis is a typical character for tumor cells. And one mechanism for many therapeutic drugs was to induce tumor cell apoptosis by caspase-8-regulated plasma membrane extrinsic pathway or caspase-9-regulated cell damage intrinsic pathway. As shown in Fig. 2c, the expression of full length pro-caspase-8 decreased dramatically after treated with 20 or 60 μg/ml rTCS for 24 h compared to control. And the anti-apoptosis molecule Bcl-2 decreased too. But cleaved caspase-8 and Bax was up-regulated significantly after 24 h. These data demonstrated that rTCS activated caspase-8-mediated apoptosis pathway in PC3 cells.

### rTCS suppressed tumor growth in nude mice

**Fig. 3:**
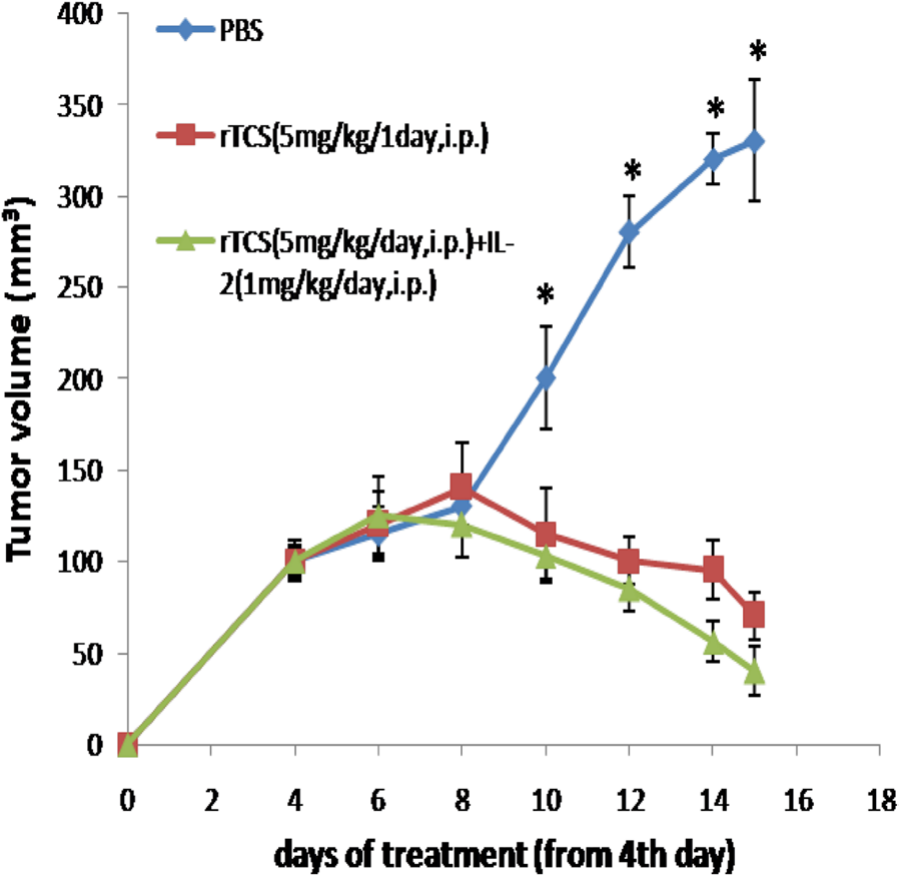
rTCS inhibits PC3 xenograft tumor growth in nude mice. Eighteen female BALB/c mice was injected with PC3 cells and divided into three groups. One group was treated with 5 mg/kg/day rTCS for 10 days. The other group was treated with rTCS (5.0 mg/kg/day) + IL-2 (1 mg/kg/day) for 10 days. In the control group, PBS was administrated. On day 15, mice were sacrificed and tumor body was weighed. *P<0.05 indicated a statistically significant difference

Similar to in vitro assay, in vivo data indicated that rTCS could suppress growth of PC3 xenograft tumor. The nude mice bearing PC3 xenograft tumor was treated with rTCS (5 mg/kg body weight, i.p. once a day for 10 days) alone or combined with IL-2 (1 mg/kg body weight, i.p. once a day for 10 days). As shown in Fig. 3, on the tenth day, the tumor volume showed an obvious reduction compared with control. On the 15th day, mice were sacrificed and tumor weight decreased significantly in both rTCS group (100 ± 22 versus 40 ± 13 mg) and rTCS/IL-2 group (100 ± 22 versus 22 ± 10 mg) compared with control. Also there was significant difference between rTCS-treated group and rTCS/IL-2-treated group.

## Discussion

Prostate cancer ranks as one of the most common and severe cancer in men with increasing incidence as well as high risk of metastasis and relapse. Medicinal plants are rich sources of biologically active natural products for drug development. It was reported that trichosanthin regulated immune responses and induced apoptosis in several kinds of tumor cells. However, its effects on prostate cancer are rarely studied. Prostate cancer is the most common malignancy for men worldwide. In the United States, there were an estimated 220,800 new cases of prostate cancer and 27,540 deaths in 2015, representing 26 % of new cancer cases and 9 % of male cancer death [21]. This makes prostate cancer the second leading cause of cancer death in men. Although the disease can potentially be cured when localized, metastatic prostate cancer remains incurable. Treatment of localized prostate cancer is usually based on surgery and/or radiation therapy. However, even after definitive local therapy, approximately 30–50 % of patients will have a local or distant recurrence. Patients with metastatic prostate cancer only have a median survival of 3–7 years followed by death [22]. Treatment options for these men are limited and do not improve overall survival [23]. Novel therapies for these patients are urgently needed.

In recent years, scientists are trying to develop new drugs and new therapeutic strategy for prostate cancer. Immune-based therapy against cancer is extensively studied. The accelerated understanding of molecular mechanisms underlying prostate cancer growth and spread fueled the hope fighting against prostate cancer.

Translational and laboratory-based clinical investigations of novel drugs are in progress. The 27-kDa trichosanthin is a ribosome inactivating protein extracted from tubers of the Chinese herbal plant *Trichosanthes kirilowii Maximowicz* [24, 25]. In this study, rTCS obtained by gene-engineering technology showed significant inhibitory effect on PC3 cells growth. We also indicated that rTCS potentially activated caspase-8-regulatory pathway and induced apoptosis of PC3 cells, which was in consistent with early research [26]. Moreover, rTCS could significantly reduce the PC3 xenograft tumor weight and tumor volume in nude mice.

Nowadays combination therapy in animal model as well as in preclinical experiments holds a promising strategy [27, 28]. Interleukin IL-2 is an immune-regulatory cytokine [29, 30] and has been applied in clinical to treat a series of diseases [31, 32]. Here IL-2 was shown to improve the anti-proliferation effect of rTCS on PC3 cells. A significant synergic effect between rTCS and IL-2 was demonstrated in vitro and in vivo. The combination therapy strategy between IL-2 and rTCS may bring new hope for patients with prostate cancer.

## Conclusion

Trichosanthin was successfully expressed and purified in this study. The recombinant trichosanthin was shown to inhibit growth of prostate cancer cells PC3 both in vitro and in vivo. The caspase-8 mediated apoptosis pathway was shown to be activated by the recombinant trichosanthin. Moreover, trichosanthin was demonstrated to function synergically with interleukin-2 to inhibit the growth of PC3 cells. Our data provides a promising hope for clinical application of rTCS/IL-2 in future.
